# The host cellular immune response to cytomegalovirus targets the endothelium and is associated with increased arterial stiffness in ANCA-associated vasculitis

**DOI:** 10.1186/s13075-018-1695-8

**Published:** 2018-08-29

**Authors:** Dimitrios Chanouzas, Michael Sagmeister, Lovesh Dyall, Phoebe Sharp, Lucy Powley, Serena Johal, Jessica Bowen, Peter Nightingale, Charles J. Ferro, Matthew D. Morgan, Paul Moss, Lorraine Harper

**Affiliations:** 1 Institute of Inflammation and Ageing, College of Medical and Dental Sciences, University of Birmingham, Birmingham, B15 2TT UK; 2 Renal Unit, University Hospitals Birmingham NHS Foundation Trust, Mindelsohn Way, Edgbaston, Birmingham, B15 2TH UK; 3 Institute of Translational Medicine Birmingham, Heritage Building, Mindelsohn Way, Edgbaston, Birmingham, B15 2TH UK; 4 Institute of Clinical Sciences, College of Medical and Dental Sciences, University of Birmingham, Birmingham, B15 2TT UK; 5 Institute of Immunology and Immunotherapy, College of Medical and Dental Sciences, University of Birmingham, Birmingham, B15 2TT UK

**Keywords:** ANCA, Vasculitis, Cytomegalovirus, Inflammation, T cells, Arterial stiffness, Cardiovascular disease

## Abstract

**Background:**

Cardiovascular disease is a leading cause of death in ANCA-associated vasculitis (AAV). An expansion of CD4^+^CD28^null^ T cells is seen mainly in cytomegalovirus (CMV)-seropositive individuals and has been linked to increased cardiovascular disease risk in other conditions. The aims of this study were to phenotype CD4^+^CD28^null^ T cells in AAV with respect to their pro-inflammatory capacity and ability to target and damage the endothelium and to investigate their relationship to arterial stiffness, a marker of cardiovascular mortality.

**Methods:**

CD4^+^CD28^null^ T cells were phenotyped in 53 CMV-seropositive AAV patients in stable remission and 30 age-matched CMV-seropositive healthy volunteers by flow cytometry following stimulation with CMV lysate. The expression of endothelial homing markers and cytotoxic molecules was evaluated in unstimulated CD4^+^CD28^null^ T cells. Arterial stiffness was measured by carotid-to-femoral pulse wave velocity (PWV) in patients with AAV.

**Results:**

CD4^+^CD28^null^ T cells were CMV-specific and expressed a T helper 1 (Th1) phenotype with high levels of interferon-gamma (IFN-γ) and tumour necrosis factor-alpha (TNF-α) secretion. They also co-expressed the endothelial homing markers CX3CR1, CD49d and CD11b and cytotoxic molecules perforin and granzyme B. CD4^+^CD28^null^ T cells were phenotypically similar in patients with AAV and healthy volunteers but their proportion was almost twice as high in patients with AAV (11.3% [3.7–19.7] versus 6.7 [2.4–8.8]; *P* = 0.022). The size of the CD4^+^CD28^null^ T-cell subset was independently linked to increased PWV in AAV (0.66 m/s increase per 10% increase in CD4^+^CD28^null^ cells, 95% confidence interval 0.13–1.19; *P* = 0.016).

**Conclusion:**

The host cellular immune response to CMV leads to the expansion of cytotoxic CD4^+^CD28^null^ T cells that express endothelial homing markers and are independently linked to increased arterial stiffness, a marker of cardiovascular mortality. Suppression of CMV in AAV may be of therapeutic value in reducing the risk of cardiovascular disease.

**Electronic supplementary material:**

The online version of this article (10.1186/s13075-018-1695-8) contains supplementary material, which is available to authorized users.

## Background

Inflammation is a key factor in the pathophysiology of atherosclerosis [1, 2]. The relationship between inflammation and cardiovascular disease (CVD) is evident in patients with rheumatic disorders such as rheumatoid arthritis, systemic lupus erythematosus and ANCA-associated vasculitis (AAV), in which CVD is a leading cause of death [3–5]. Traditional risk factors do not fully explain the increased incidence of CVD seen in these conditions [6], and it is thought that inflammation and immunopathology may accelerate atherosclerosis [7].

CD4 T cells that do not express the co-stimulatory molecule CD28 (CD4^+^CD28^null^) have been implicated in vascular injury [8]. CD4^+^CD28^null^ T cells are pro-inflammatory and their proportion expands under inflammatory conditions [9–11]. They are found preferentially in unstable rather than stable atherosclerotic plaques [12], suggesting direct involvement in plaque disruption, and they have been shown in *in vitro* assays to exhibit endothelial cytotoxicity in the context of acute coronary syndrome [13] and AAV [14]. Several studies in patients with inflammatory disorders such as rheumatoid arthritis have demonstrated that expansion of CD4^+^CD28^null^ T cells is independently associated with increased incidence of CVD and cardiovascular mortality [15–19].

Loss of the co-stimulatory molecule CD28 on CD4 T cells suggests repeated exposure to a persistent antigen [20]. We and others have demonstrated that significant expansion of CD4^+^CD28^null^ T cells occurs mainly in cytomegalovirus (CMV)-seropositive individuals, and negligible or very low proportions of these cells are seen in the absence of previous CMV infection [11, 21–24]. CMV infection is widely prevalent in the general population [25], and CMV itself has been implicated in the pathogenesis of CVD [26]. CMV infects endothelial and smooth muscle cells where it is able to persist during latency [27]. Infection with CMV is associated with impaired vascular function [28], high blood pressure [29], increased arterial stiffness [30] and cardiovascular mortality [26]. Furthermore, a recent meta-analysis demonstrated that CMV infection is associated with a 22% increased relative risk for CVD in the general population [31].

The aims of this study were to characterise the phenotype of CD4^+^CD28^null^ T cells in AAV, with respect to their pro-inflammatory capacity and ability to target and damage the endothelium, and to determine whether expansion of this cell subset is associated with arterial stiffness, a marker of cardiovascular mortality.

## Methods

### Study population

Fifty-three CMV-seropositive patients with AAV in stable remission were recruited from the vasculitis clinic at University Hospitals Birmingham NHS Foundation Trust (Birmingham, UK), and 30 age-matched CMV-seropositive healthy volunteers (HVs) were enrolled from the 1000 Elders Cohort (courtesy of Professor Janet Lord, University of Birmingham, UK) and patient household contacts. CD4^+^CD28^null^ T-cell percentage and phenotype were assessed in all participants. Arterial stiffness was measured in patients with AAV.

Patients were eligible for inclusion if they had a documented diagnosis of AAV and were in stable remission for at least 6 months, on maintenance immunosuppression with a maximum of two agents, and seropositive for CMV (anti-CMV IgG detected in peripheral blood). Exclusion criteria were estimated glomerular filtration rate of less than 15 mL/minute per 1.73 m^2^, B cell–depleting therapy within 12 months or T cell–depleting therapy within 6 months, presence of other chronic infection (HIV, hepatitis B, hepatitis C, or tuberculosis) and treatment with anti-CMV therapies within the previous month.

Thirty-eight of 53 patients with AAV were participants in the ‘Cytomegalovirus modulation of the immune system in ANca-associated VASculitis’ (CANVAS) clinical trial, a proof-of-concept open-label randomised trial of valaciclovir, or no additional treatment, in CMV-seropositive AAV patients in remission [32]. All immune and arterial stiffness assessments reported here were conducted at baseline prior to commencement of valaciclovir.

The study was approved by the Research Ethics Committee of Yorkshire and the Humber (UK). Written informed consent was obtained from all participants.

### Blood collection

Up to 50 mL of peripheral blood was obtained and processed within a maximum of 5 hours following venepuncture. Plasma was isolated by centrifugation and cryopreserved at −80 °C. Peripheral blood mononuclear cells (PBMCs) were isolated from heparinised blood by density gradient centrifugation, used immediately in stimulation experiments with CMV lysate to identify cytokine-producing T cells, or cryopreserved in liquid nitrogen.

Cells for flow cytometry experiments were acquired on a BD LSR II Flow Cytometer and analysed by using FACS DIVA Software Version 8.0 (BD, Franklin Lakes, NJ, USA). Monoclonal antibodies used for flow cytometry experiments are listed in Table S1 of Additional file 1. Gating strategies are shown in Figure S1 and S2 of Additional file 1.

**Table 1:** Participant baseline characteristics

	AAV (*n* = 53)	HV (*n* = 30)
Age, years	69.0 [62.8–75.3]	70.5 [66.8–74.0]^*^
Gender, male:female	35:18	14:16^*^
ANCA specificity, PR3:MPO	34:18	–
AAV disease chronicity, years	6.0 [3.2–12.0]	–
Renal function eGFR, mL/min per 1.73 m^2^	53 [21]	–
Urine albumin-to-creatinine ratio, mg/mmol	4.4 [1.4–9.9]	–
Steroids, n (%)	39 (73.6)	–
Mycophenolate mofetil, n (%)	14 (26.4)	–
Azathioprine, n (%)	19 (35.8)	–
No current immunosuppression, n (%)	4 (7.5)	–
Ever smoker, n (%)	26 (49.1)	–
Diabetes mellitus, n (%)	11 (20.8)	–
On statin treatment, n (%)	29 (54.7)	

Data are displayed as median [interquartile range] apart from renal function displayed as mean [standard deviation]. Immunosuppressive treatment refers to number and percentage of patients on the respective immunosuppressive agent at the time of study entry.

^*^Comparison between ANCA-associated vasculitis (AAV) and healthy volunteers (HV): age (Mann–Whitney *U* test, *P* = 0.557), gender (Fisher’s exact, *P* = 0.106)

Abbreviations: *eGFR* estimated glomerular filtration rate, *MPO* myeloperoxidase, *PR3* proteinase 3

**Fig. 1:**
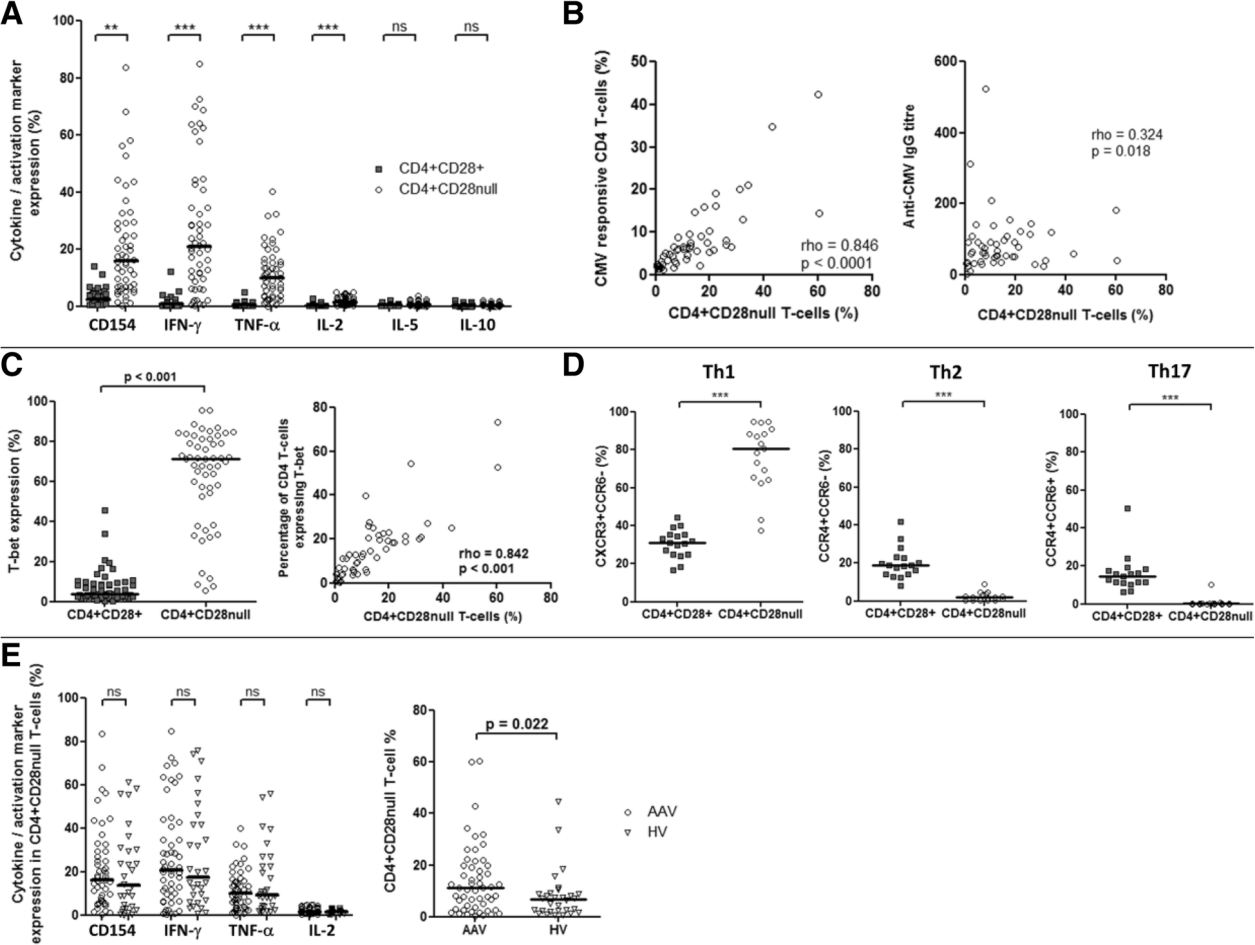
CD4^+^CD28^null^ T cells are cytomegalovirus (CMV)-responsive T helper 1 (Th1) pro-inflammatory T cells. **a** Following stimulation with CMV lysate, a greater proportion of CD4^+^CD28^null^ T cells (open circles) expressed the activation marker CD154 and secreted interferon-gamma (IFN-γ), tumour necrosis factor-alpha (TNF-α) and interleukin-2 (IL-2) compared with CD4^+^CD28^+^ T cells (grey squares) (ANCA-associated vasculitis [AAV], *n* = 53). **b** CD4^+^CD28^null^ T-cell percentage correlated with the size of the total CD4 CMV response and with the anti-CMV IgG antibody titre (AAV, *n* = 53). **c** A greater proportion of CD4^+^CD28^null^ T cells expressed the Th1 transcription factor T-bet compared with CD4^+^CD28^+^ T cells and CD4^+^CD28^null^ T-cell percentage correlated with the percentage of CD4 T cells expressing T-bet (AAV, n = 53). **d** CD4^+^CD28^null^ T cells exhibited a Th1 lineage chemokine receptor pattern rather than Th2 or Th17 (AAV, *n* = 17). **e** There was no difference in the cytokine expression profile between AAV (n = 53, open circles) and healthy volunteers (HV) (*n* = 30, open triangles), but patients with AAV contained larger expansions of CD4^+^CD28^null^ T cells. **P < 0.01, ***P < 0.001. All bars are medians. Abbreviation: *ns* not significant

**Fig. 2:**
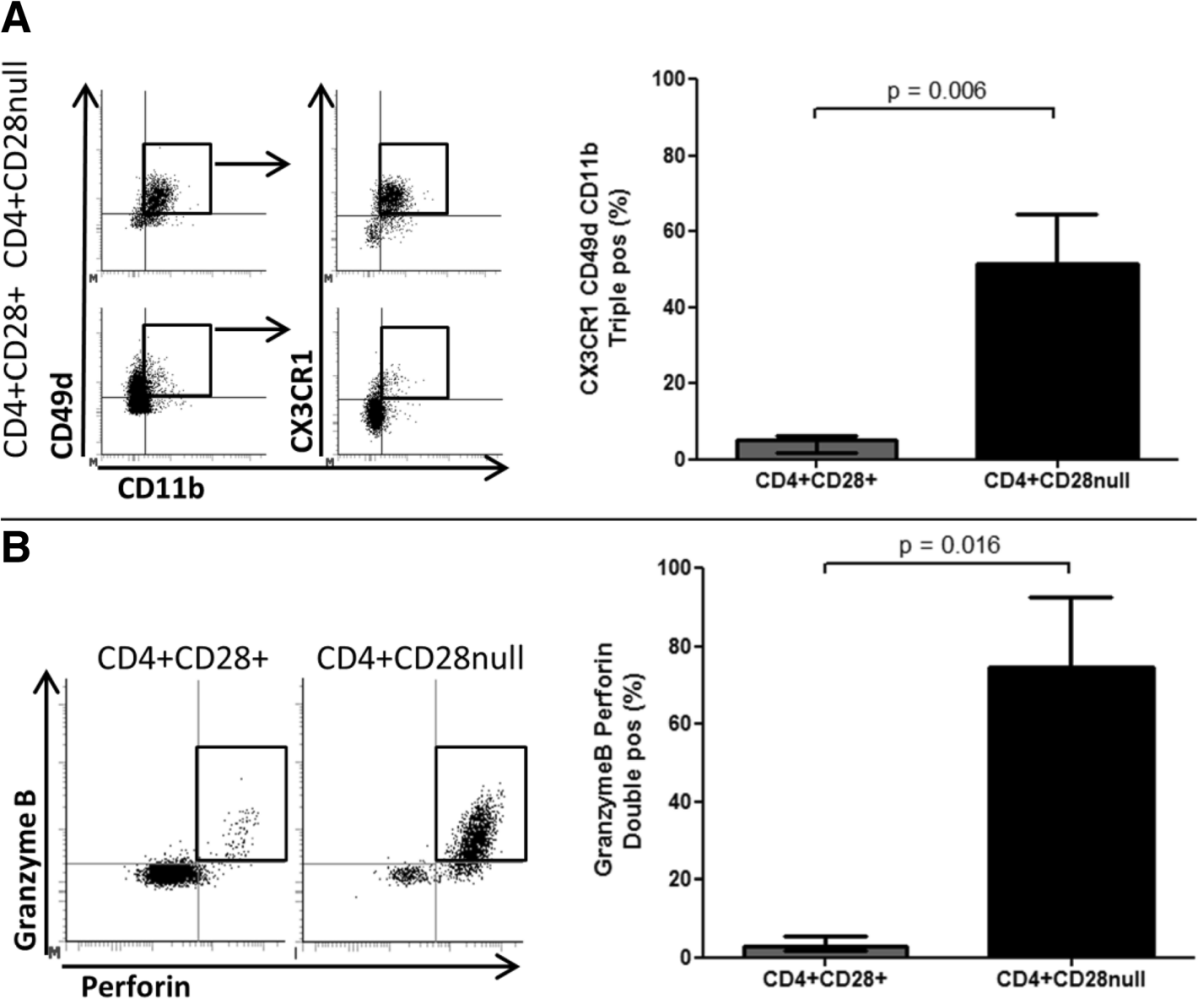
CD4^+^CD28^null^ T cells are endothelial homing, cytotoxic T cells. Unstimulated CD4 T cells from patients with ANCA-associated vasculitis (AAV) (*n* = 10) were stained for expression of endothelial homing receptors CX3CR1, CD49d and CD11b and intracellular cytotoxic molecules perforin and granzyme B. **a** Representative staining and summary data for unstimulated CD4 T cells gated on CD4^+^CD28^null^ (top two flow cytometry plots) and CD4^+^CD28^+^ T cells (bottom two flow cytometry plots). Sequential gating was performed as follows: CD49d and CD11b to identify CD49d CD11b double-positive cells, followed by CX3CR1 to identify CD49d CD11b CX3CR1 triple-positive cells. **b** Representative staining and summary data for unstimulated CD4 T cells gated on CD4^+^CD28^+^ and CD4^+^CD28^null^ T cells showing cells double-positive for perforin and granzyme B

### Enumeration of peripheral blood CD4^+^CD28^null^ T cells

Whole blood was stained with anti-CD3, anti-CD4 and anti-CD28 monoclonal antibodies to determine CD4^+^CD28^null^ T-cell percentage. Quality control was achieved by using a positive control (Cytofix CD4 Normal Range Positive Control; Cytomark, Caltag Medsystems, Buckingham, UK) with a validated acceptance range for CD3^+^CD4^+^ percentage and a fluorescence minus one control to aid CD28 gating. These were assayed with every analytical run.

### Peripheral blood mononuclear cell stimulation

To identify CMV lysate-stimulated cytokine-expressing T cells, 0.5–1 × 10^6^ PBMCs were re-suspended in supplemented medium—(RPMI), 10% foetal calf serum; sterile filtered and heat inactivated (Sigma-Aldrich, St. Louis, MO, USA), 1% penicillin/streptomycin (P/S) (Gibco, Thermo Fisher Scientific, Waltham, MA, USA—overnight for 16 (±2) hours at 37 °C, 5% CO_2_, in the presence of monensin (2 μmol/L) and phycoerythrin-conjugated anti-CD154 monoclonal antibody as previously described [33]. Cells were stimulated with CMV lysate (1:100) prepared from CMV strain AD169-infected human foetal foreskin fibroblasts. Unstimulated cells served as controls. Following overnight incubation, cells were stained with eFluor-506 viability dye (eBioscience, Thermo Fisher Scientific) for 30 min at 4 °C, washed with phosphate-buffered saline and flow cytometry buffer, and co-stained with saturating amounts of anti-CD3, anti-CD4 and anti-CD28 antibodies for 30 min at 4 °C before washing with flow cytometry buffer. Cells were fixed and permeabilised with an intracellular flow cytometry staining kit (eBioscience) and stained for 30 min at 4 °C with saturating amounts of anti-interferon-gamma (anti-IFN-γ), anti-tumour necrosis factor-alpha (anti-TNF-α), anti-interleukin-2 (anti-IL-2), anti-IL-5, anti-IL-10 and anti-T-bet monoclonal antibodies before washing with flow cytometry buffer.

### Identification of Th1-, Th2- and Th17-skewed subsets

The expression of chemokine receptors on unstimulated CD4^+^CD28^null^ T cells from 17 patients with AAV was defined by staining whole blood with anti-CXCR3, anti-CCR4, anti-CCR6, anti-CD3, anti-CD4 and anti-CD28 monoclonal antibodies. T helper 1 (Th1)-skewed CD4 T cells were identified as CXCR3^+^CCR6^−^, Th2-skewed as CCR4^+^CCR6^−^ and Th17-skewed as CCR4^+^CCR6^+^ [34].

### Identification of endothelial homing receptors and cytotoxic molecules

In order to phenotype unstimulated CD4^+^CD28^null^ T cells with respect to their expression of endothelial homing receptors and cytotoxic molecules, cryopreserved PBMCs from 10 patients with AAV were stained with Fixable Viability dye eFluor-506 as described above; co-stained with anti-CD3, anti-CD4, anti-CD28, anti-CX3CR1, anti-CD49d and anti-CD11b antibodies; and fixed and permeabilised as already described, followed by intracellular staining with anti-perforin and anti-granzyme B antibodies.

### Measurement of soluble markers of inflammation

Soluble markers of inflammation (IL-2, TNF-α, IFN-γ, IL-10, IL-17A, IL-6 and highly sensitive C-reactive protein) were measured in plasma by Luminex array (ProcartaPlex, eBioscience) in accordance with the instructions of the manufacturer, read on a Bio-Rad Luminex 200 instrument (Bio-Rad, Hercules, CA, USA) and analysed by using ProcartaPlex Analyst 1.0 Software (eBioscience).

### Determination of anti-CMV IgG titre

Plasma anti-CMV IgG titre was assayed by using an enzyme-linked immunosorbent assay as previously described [30]. CMV seropositivity was defined as an anti-CMV IgG titre of more than 10 units.

### Arterial stiffness measurement

Arterial stiffness was estimated by measuring carotid-to-femoral pulse wave velocity (PWV) using the non-invasive, non-operator-dependent Vicorder system (Skidmore, Bristol, UK) that employs a volume displacement method, as previously described [32]. Briefly, the patient was allowed to rest for 5 min prior to inflating a 100-mm-wide cuff on the non-dominant arm to measure peripheral blood pressure. A 30-mm-wide cuff was then placed on the neck at the level of the carotid artery and a 100-mm-wide cuff placed around the proximal thigh. The distance between the mid-clavicular point and the mid-point of the thigh cuff, the aortic path length, was measured with the patient supine. With the patient at a supine 30° head-tilt position, the cuffs were inflated to 60 mm Hg. The Vicorder instrument uses the resultant oscillometric signal to extract the pulse waveforms and pulse transit time to calculate carotid-to-femoral PWV. The mean value of three consistent readings was used for subsequent analysis.

### Statistical analysis

Continuous variables were summarised as medians and quartiles (unless stated otherwise) and categorical data as counts and percentages. Groups were compared with the Mann–Whitney test, the Kruskal–Wallis and Dunn’s multiple comparison test, and the chi-squared or Fisher’s exact test. Associations were assessed with Spearman’s rank correlation. Univariable and multivariable linear regression models were constructed for variables associated with PWV. Analyses were undertaken by using SPSS Statistics Version 21 (IBM, Armonk, NY, USA) and Prism Version 5 (GraphPad, La Jolla, CA, USA) and were two-tailed; a *P* value of less than 0.05 was considered significant.

## Results

Baseline characteristics of study participants are shown in Table 1.

### CD4^+^CD28^null^ T cells are CMV-specific and display a pro-inflammatory phenotype

We initially undertook a phenotypic analysis of CD4^+^CD28^null^ T cells from 53 CMV-seropositive AAV patients in stable remission. PBMCs were stimulated *in vitro* with CMV lysate, and the proportion of CMV-specific cells within the CD4^+^CD28^null^ and CD4^+^CD28^+^ subfractions was determined. A greater proportion of CMV-specific T cells was observed within the CD4^+^CD28^null^ population as determined by expression of the activation marker CD154 and cytokine expression: IFN-γ, TNF-α and IL-2 (Fig. 1a). Neither cell type expressed significant amounts of IL-5 or IL-10.

The percentage of CD4^+^CD28^null^ T cells was strongly correlated with the total CMV-specific CD4^+^ response (rho = 0.846, *P* <0.001; Fig. 1b), indicating that the size of the CD4^+^CD28^null^ T-cell accumulation is a good measure of the impact of CMV infection on the CD4 compartment in this population. CD4^+^CD28^null^ T-cell percentage also correlated with the humoral response to CMV, anti-CMV IgG titre (rho = 0.324, *P* = 0.018; Fig. 1b).

As well as expressing IFN-γ and TNF-α, the majority of CD4^+^CD28^null^ T cells expressed the Th1 lineage transcription factor T-bet (Fig. 1c) and a Th1-skewed chemokine receptor profile (CXCR3^+^CCR6^−^) (Fig. 1d). In contrast, the chemokine receptor staining profile that identifies Th2-skewed (CCR4^+^CCR6^−^) and Th17-skewed (CCR4^+^CCR6^+^) cells was not seen on CD4^+^CD28^null^ T cells. Indeed, CD4^+^CD28^null^ T cells comprised the majority of the Th1 compartment (median 51.6%, 30.0–77.3). In addition, the percentage of CD4^+^CD28^null^ T cells was strongly correlated with the overall size of the Th1 compartment (Fig. 1c), indicating that the size of the CD4^+^CD28^null^ T-cell expansion in CMV-seropositive AAV patients determines the relative proportion of Th1 cells, a subset known to exert a strong pro-atherosclerotic effect [35].

There was no difference between AAV patients and age-matched CMV-seropositive HVs in the induction of CD154 expression on CD4^+^CD28^null^ T cells or their cytokine expression profile following CMV lysate stimulation (Fig. 1e). However, patients with AAV had larger expansions of CD4^+^CD28^null^ T cells compared with HVs (11.3% [3.7–19.7] versus 6.7 [2.4–8.8]; *P* = 0.022).

### CD4^+^CD28^null^ T cells are endothelial homing cytotoxic T cells

IFN-γ and TNF-α activate endothelial cells and increase surface expression of chemokines and adhesion molecules such as IFN-γ–inducible protein-10 (IP-10), fractalkine, vascular adhesion molecule-1 (VCAM-1) and intercellular adhesion molecule-1 (ICAM-1) [36]. Having identified high levels of expression of the IP-10 receptor CXCR3 on the surface of CD4^+^CD28^null^ T cells, we evaluated the surface expression of the receptors for fractalkine, VCAM-1 and ICAM-1 (CX3CR1, CD49d and CD11b) on unstimulated PBMCs from 10 patients with AAV. Co-expression of all three endothelial homing receptors was significantly more common on CD4^+^CD28^null^ compared with CD4^+^CD28^+^ T cells (51.6% [42.7–64.4] versus 5.0 [1.7–6.3]; *P* = 0.006; Fig. 2a). In addition, the majority of unstimulated CD4^+^CD28^null^ T cells contained intracellular stores of both perforin and granzyme B (74.5%, 63.5–92.7; Fig. 2b), suggesting that they have the capacity to target and lyze endothelial cells.

### The size of the CD4^+^CD28^null^ T-cell expansion is independently associated with increased arterial stiffness in AAV

**Fig. 3:**
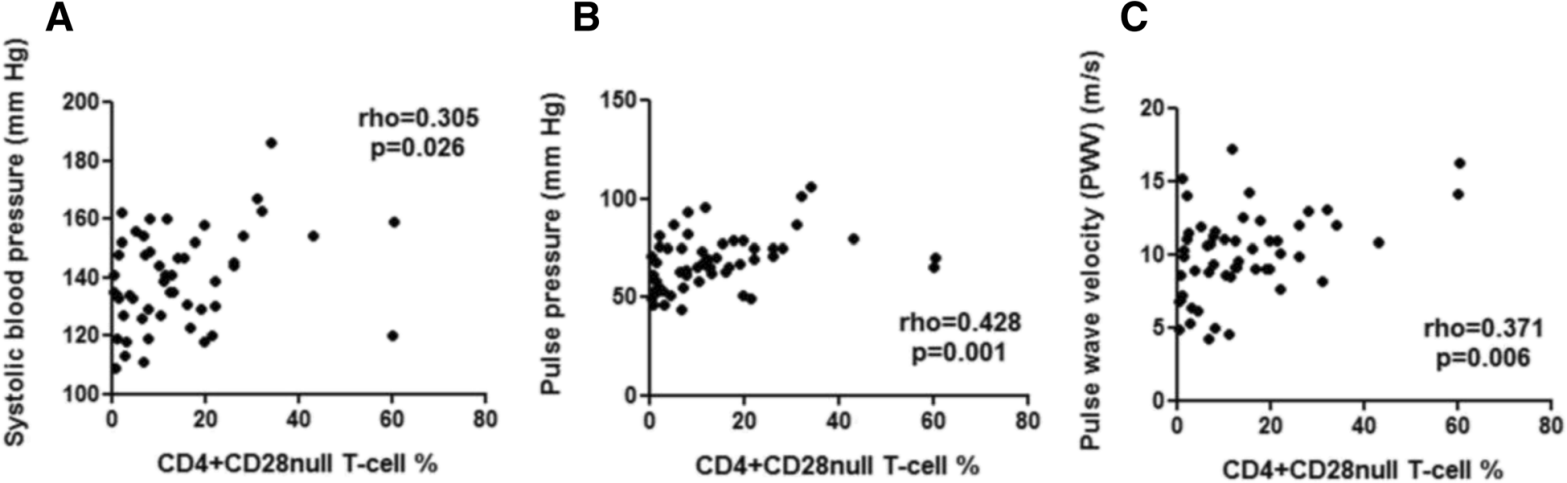
CD4^+^CD28^null^ T-cell expansion correlates with increased arterial stiffness and blood pressure parameters. CD4^+^CD28^null^ T-cell percentage correlated with increased systolic blood pressure (**a**), pulse pressure (**b**), a surrogate marker of arterial stiffness, and carotid-to-femoral pulse wave velocity (**c**) (n = 53)

To determine whether the size of the CD4^+^CD28^null^ T-cell expansion is associated with arterial stiffness, as a clinical marker of cardiovascular pathology, carotid-to-femoral PWV was measured in the patients with AAV. The CD4^+^CD28^null^ T-cell percentage was found to be significantly correlated with increased systolic blood pressure (rho = 0.305, *P* = 0.026), pulse pressure (rho = 0.428, *P* = 0.001) and PWV (rho = 0.371, *P* = 0.006; Fig. 3).

On univariable analysis, age, percentage of CD4^+^CD28^null^ T cells, plasma concentration of TNF-α, and blood pressure parameters (Table 2) were associated with increased PWV. In contrast, we did not observe an association between anti-CMV IgG titre and PWV.

**Table 2:** Variables associated with pulse wave velocity on univariable analysis

Variable	Univariable analysis		
	R^2^	Regression coefficient [95% CI]	*P* value
Age, years	0.183	0.131 [0.053, 0.208]	0.001
Gender	0.004	0.393 [− 1.313, 2.100]	0.645
eGFR, mL/min per 1.73 m^2^	0.026	− 0.023 [− 0.062, 0.017]	0.252
Urinary albumin-to-creatinine ratio, mg/mmol	0.056	0.019 [−0.003, 0.041]	0.088
Ever smoker	0.017	−0.759 [−2.364, 0.847]	0.347
Presence of diabetes	0.002	0.291 [−1.704, 2.286]	0.771
On statin treatment	0.044	1.208 [−0.383, 2.799]	0.134
Pulse pressure, mm Hg	0.163	0.084 [0.030, 0.137]	0.003
Mean arterial pressure, mm Hg	0.102	0.088 [0.015, 0.161]	0.020
Systolic blood pressure, mm Hg	0.191	0.079 [0.033, 0.124]	0.001
Diastolic blood pressure, mm Hg	0.027	0.056 [−0.039, 0.150]	0.243
CD4^+^CD28^null^ T-cell proportion (%), per 10% increase	0.182	0.912 [0.368, 1.455]	0.001
C-reactive protein, mg/mL	0.008	−0.234 [− 0.958, 0.490]	0.520
IL-2, pg/mL	0.066	0.007 [0.000, 0.015]	0.065
IFN-γ, pg/mL	0.036	−0.004 [− 0.010, 0.002]	0.179
IL-10, pg/mL	0.025	0.089 [−0.068, 0.245]	0.260
IL-6, pg/mL	0.004	0.002 [−0.007, 0.011]	0.649
TNF-α, pg/mL	0.083	0.015 [0.001, 0.029]	0.039
IL-17, pg/mL	0.015	−0.003 [−0.011, 0.004]	0.389

All variables with a *P* value of less  than 0.1 on univariable analysis were included in the multivariable model shown in Table 3

Abbreviations: *CI* confidence interval, *eGFR* estimated glomerular filtration rate, *IL* interleukin, *TNF-α* tumour necrosis factor-alpha

To account for confounding factors, we constructed a multivariable linear regression model including all variables that were associated with PWV on univariable analysis with a *P* value of less than 0.1 (Tables 2 and 3). This demonstrated that the percentage of CD4^+^CD28^null^ T cells associated with increased arterial stiffness independently of age, proteinuria, peripheral mean arterial blood pressure, and plasma concentration of TNF-α. There was a 0.66 m/s [95% confidence interval 0.13–1.19] increase in PWV for every 10% increase in CD4^+^CD28^null^ T cells (*P* = 0.016; Table 3). This relationship did not change when systolic blood pressure or pulse pressure was substituted for mean arterial blood pressure, and the size of the CD4^+^CD28^null^ T-cell expansion remained independently associated with increased PWV.

**Table 3:** Multivariable linear regression model for pulse wave velocity (meters per second)

Variable	Univariable analysis		Multivariable analysis	
	Regression coefficient [95% CI]	*P* value	Regression coefficient [95% CI]	*P* value
CD4^+^CD28^null^ T-cell percentage, per 10% increase	0.912 [0.368, 1.455]	0.001	0.663 [0.132, 1.194]	0.016
Age, years	0.131 [0.053, 0.208]	0.001	0.080 [0.006, 0.155]	0.035
Proteinuria (urinary ACR), mg/mmol	0.019 [−0.003, 0.041]	0.088	0.013 [−0.007, 0.033]	0.196
Mean arterial pressure, mm Hg	0.088 [0.015, 0.161]	0.020	0.053 [−0.016, 0.122]	0.128
TNF-α, pg/mL	0.015 [0.001, 0.029]	0.039	0.010 [−0.002, 0.022]	0.086

Variables with *P* value of less than 0.1 on univariable analysis (Table 2) were included in the model. In order to avoid collinearity, only one blood pressure parameter and either plasma concentration of tumour necrosis factor-alpha (TNF-α) or interleukin-2 (IL-2) were added at each iteration of the model. This table shows the final model with mean arterial pressure (MAP) and TNF-α. The size of the CD4^+^CD28^null^ T-cell expansion remained independently associated with pulse wave velocity with negligible impact on the model characteristics when pulse pressure or systolic blood pressure was substituted for MAP and when plasma concentration of IL-2 was substituted for TNF-α

R value: 0.635, R^2^: 0.404

Abbreviations: *ACR* albumin-to-creatinine ratio, *CI* confidence interval

## Discussion

Our findings demonstrate that the host cellular immune response to CMV is associated with the expansion of a subset of pro-inflammatory, endothelial homing, cytotoxic CD4^+^CD28^null^ T cells in patients with AAV. Furthermore, the size of this expansion is independently linked to increased arterial stiffness, a marker of cardiovascular mortality [37]. This is significant in AAV as cardiovascular disease is a leading cause of death in this patient group.

Our data provide further insight into the properties of CD4^+^CD28^null^ T cells and their capacity to cause vascular damage of relevance to AAV. We identified CD4^+^CD28^null^ T cells in AAV to be Th1-skewed pro-inflammatory T cells that produce IFN-γ and TNF-α in response to stimulation with CMV lysate. These findings are in keeping with previous reports in HVs [38] as well as in AAV where CD4^+^CD28^null^ T cells have been shown to be a major source of IFN-γ and TNF-α [39]. Th1 T cells are recognised as important players in the atherosclerotic process with Th1-driven responses exerting detrimental effects [40]. Mice deficient in T-bet, the master regulator of the Th1 transcriptional response, are relatively protected from the development of atherosclerotic lesions [35], and both T-bet and IFN-γ are essential in the generation of angiotensin II–mediated vascular dysfunction [41]. We observed that the CD4 Th1 compartment in AAV CMV-seropositive patients was made up mostly of CD4^+^CD28^null^ T cells and that up to 94% of Th1 cells were CD4^+^CD28^null^. Taken together, our findings indicate that CMV exerts a powerful influence on the shape and magnitude of the Th1 repertoire.

IFN-γ and TNF-α cytokines mediate inflammation in blood vessel walls through disruption of endothelial junctions and induction of chemokine and adhesion molecule expression on vascular endothelium. This promotes the recruitment and adherence of lymphocytes and monocytes on the inflamed endothelium and facilitates leukocyte transmigration [36]. In our phenotypic analysis, CD4^+^CD28^null^ T cells were found to express the chemokine receptors CX3CR1, CD49d, CD11b and CXCR3 that are able to bind their respective adhesion molecule ligands fractalkine, VCAM-1, ICAM-1 and IP-10 on activated endothelial cells [42, 43]. They also co-expressed the cytolytic granules granzyme B and perforin, as previously found by others [44], suggesting that they act as cytotoxic effector cells. The endothelium is an important site for CMV infection [27] and as such endothelial targeting by CMV-specific T cells would be expected to have the capacity to suppress viral reactivation but might also contribute to vascular damage.

Consistent with these observations, our findings revealed that the size of the CD4^+^CD28^null^ T-cell expansion in patients with AAV was independently associated with increased arterial stiffness. This is contrary to a recent report in patients with AAV where the size of the CD4^+^CD28^null^ T-cell expansion was found not to be related to arterial stiffness [45]. However, the AAV cohort within that study included a substantial proportion of CMV-seronegative patients and only 24 patients were CMV-seropositive. Given that significant expansion of CD4^+^CD28^null^ T cells is seen mainly in CMV-seropositive individuals, this may explain the discrepancy between our findings and those of Slot *et al*. [45]. Furthermore, our data are in agreement with several published studies reporting associations in other patient groups such as rheumatoid arthritis [15] and chronic kidney disease [18], between CD4^+^CD28^null^ T cells and markers of atherosclerotic damage.

Previous work by our group has shown that CMV-seropositive patients with chronic kidney disease have stiffer arteries compared with CMV-seronegative chronic kidney disease patients [30]. In the present study, we observed that expansion of CD4^+^CD28^null^ T cells in CMV-seropositive AAV patients is associated with increased arterial stiffness in that carotid-to-femoral PWV increases by 0.66 m/s for every 10% increase in the size of the CD4^+^CD28^null^ T-cell subset. Such an effect size on PWV is greater than the impact of smoking on arterial stiffness [46] and roughly equivalent to 10 years’ worth of ageing [47]. In contrast, we found no correlation between the humoral response to CMV, measured by the anti-CMV IgG titre, and arterial stiffness. Based on our data, we propose that the host cellular immune response to CMV is directly involved in the development of cardiovascular pathology and that this is driven by the expansion of the pro-inflammatory endothelial homing CD4^+^CD28^null^ T-cell subset. However, it should be noted that our study was cross-sectional in nature and therefore cannot definitively confirm a longitudinal increase in arterial stiffness driven by CD4^+^CD28^null^ T cells.

It is likely that expansion of CD4^+^CD28^null^ T cells itself is driven by asymptomatic subclinical reactivation of CMV. Viral reactivation is more likely to occur in an inflammatory milieu, and TNF-α has recently been shown to reverse the transcriptional silencing that maintains CMV latency, leading to lytic reactivation [48]. We recently observed that subclinical CMV reactivation occurs in over a quarter of patients with AAV in remission within a 12-month period [49], indicating that CMV reactivation is a frequent event in this patient group. This could explain our finding that CD4^+^CD28^null^ T-cell expansions are almost twice as large in AAV patients compared with age-matched HVs. Subclinical CMV reactivation is likely to be even higher during the acute phase of AAV, when patients are exposed to high levels of systemic inflammation and intensive immunosuppressive treatment. As viral reactivation is expected to act as a potent stimulus for boosting the CMV-specific cellular immune response, anti-viral therapy could act to suppress CMV-specific immune responses and potentially reduce cardiovascular damage. Anti-viral therapy in this context would suppress all herpes viruses. This could be advantageous as recent evidence suggests that concomitant CMV and Epstein–Barr virus infection may be associated with increased expansion of CD4^+^CD28^null^ T cells in AAV [50].

## Conclusions

In summary, the results presented here support a mechanism for vascular damage secondary to expansion of pro-inflammatory CMV-specific T cells that target the endothelium and are independently linked to arterial stiffness. Our data suggest that suppression of CMV may hold therapeutic potential for patients with AAV, lending support to the design of further studies aiming to determine whether CMV suppression might reduce expansion of CD4^+^CD28^null^ T cells, ameliorating surrogate markers of atherosclerotic damage, and ultimately reducing risk of cardiovascular disease, the leading cause of death in this patient group.

## Additional file

Additional file 1: **Table S1.** Antibodies used for flow cytometric analysis. **Figure S1.** Gating strategy for whole blood staining. **Figure S2.** Gating strategy for cytomegalovirus (CMV) lysate-stimulated peripheral blood mononuclear cells (PBMC). (DOCX 687 kb)

## Abbreviations

AAV: ANCA-associated vasculitis
ANCA: Anti-neutrophil cytoplasmic antibody
CMV: Cytomegalovirus
CVD: Cardiovascular disease
CX3CR1: CX3C chemokine receptor 1
HV: Healthy volunteer
ICAM-1: Intercellular adhesion molecule 1
IFN-γ: Interferon-gamma
IL: Interleukin
IP-10: IFN-gamma-inducible protein 10
PBMC: Peripheral blood mononuclear cell
PWV: Pulse wave velocity
Th: T helper
TNF-α: Tumour necrosis factor-alpha
VCAM-1: Vascular cell adhesion molecule 1
