# Supplementary material for: The host cellular immune response to cytomegalovirus targets the endothelium and is associated with increased arterial stiffness in ANCA-associated vasculitis
Source: Arthritis Res Ther. 2018 Aug 29;20:194. doi: 10.1186/s13075-018-1695-8 (PMC6116544; doi:10.1186/s13075-018-1695-8)
Supplement: Supplementary file 1 — Table S1. Antibodies used for flow cytometric analysis. Figure S1. Gating strategy for whole blood staining. Figure S2. Gating strategy for cytomegalovirus (CMV) lysate-stimulated peripheral blood mononuclear cells (PBMC). (DOCX 687 kb) [file 13075_2018_1695_MOESM1_ESM.docx]

**Additional file**

Table S1 Antibodies used for flow cytometric analysis

| Marker | Fluorochrome | Isotype | Clone | Concentration  μL / test | Manufacturer |
| --- | --- | --- | --- | --- | --- |
| CD3 | Brilliant Violet 650 | Mouse IgG2a, κ | OKT3 | 12 μg/mL  2 μL | Biolegend |
| CD4 | Brilliant Violet 605 | Mouse IgG2b, κ | OKT4 | 100 μg/mL  2 μL | Biolegend |
| CD28 | eFluor 450 | Mouse IgG1, κ | CD28.2 | 25 μg/mL  3 μL | eBioscience |
| CXCR3 | PE-Dazzle 594 | Mouse IgG1, κ | G025H7 | 150 μg/mL  3 μL | Biolegend |
| CCR4 | PE-Cy7 | Mouse IgG1, κ | L291H4 | 200 μg/mL  5 μL | Biolegend |
| CCR6 | PerCP-Cy5.5 | Mouse IgG2b, κ | G034E3 | 100 μg/mL  5 μL | Biolegend |
| CD154 | PE | Mouse IgG1, κ | 24-31 | 100 μg/mL  5 μL | eBioscience |
| IFN-γ | PE-CF594 | Mouse IgG1, κ | B27 | 3 μL | BD |
| TNF-α | Alexa Fluor 700 | Mouse IgG1, κ | MAb11 | 50 μg/mL  3 μL | eBioscience |
| IL-2 | PerCP eFLuor 710 | Mouse IgG2a, κ | MQ1-17H12 | 12 μg/mL  3 μL | eBioscience |
| IL-10 | PE-Cy7 | Rat IgG1, κ | JES3-9D7 | 20 μg/mL  5 μL | Biolegend |
| IL-5 | APC | Rat IgG1, κ | TRFK5 | 0.2 mg/mL  5 μL | Biolegend |
| T-bet | FITC | Mouse IgG1, κ | 4B10 | 0.5 mg/mL  2 μL | Biolegend |
| CD49d | PE-Dazzle 594 | Mouse IgG1, κ | 9F10 | 5 μL | Biolegend |
| CD11b | Brilliant Violet 650 | Mouse IgG1, κ | ICRF44 | 5 μL | Biolegend |
| CX3CR1 | PerCP-Cy5.5 | Rat IgG2b, κ | 2A9-1 | 5 μL | Biolegend |
| Perforin | FITC | Mouse IgG2b, κ | dG9 | 5 μL | Biolegend |
| Granzyme B | Alexa Fluor 647 | Mouse IgG1, κ | GB11 | 5 μL | Biolegend |


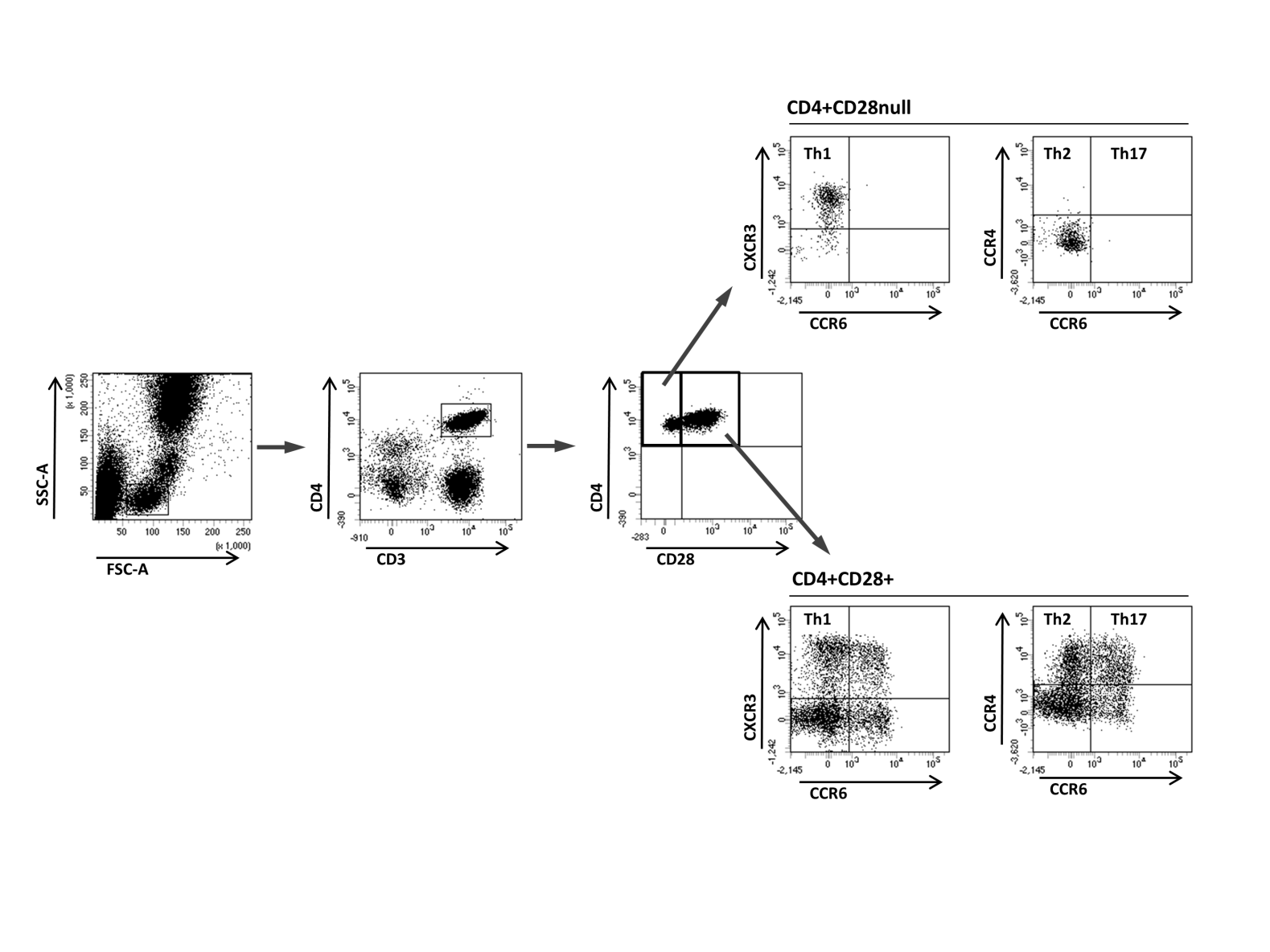


**Figure S1 Gating strategy for whole blood staining**

Whole blood was stained with monoclonal antibodies as detailed in the methods section. Sequential gating was performed as follows: lymphocyte gate according to forward scatter (FSC-A) and side scatter (SSC-A) parameters, CD3+CD4+ gate, CD28 gate to identify CD4+CD28null and CD4+CD28+ T-cells. Additional staining was performed in whole blood from 17 AAV patients to identify Th1-skewed (CXCR3+CCR6-), Th2-skewed (CCR4+CCR6-) and Th17-skewed (CCR4+CCR6+) subsets.


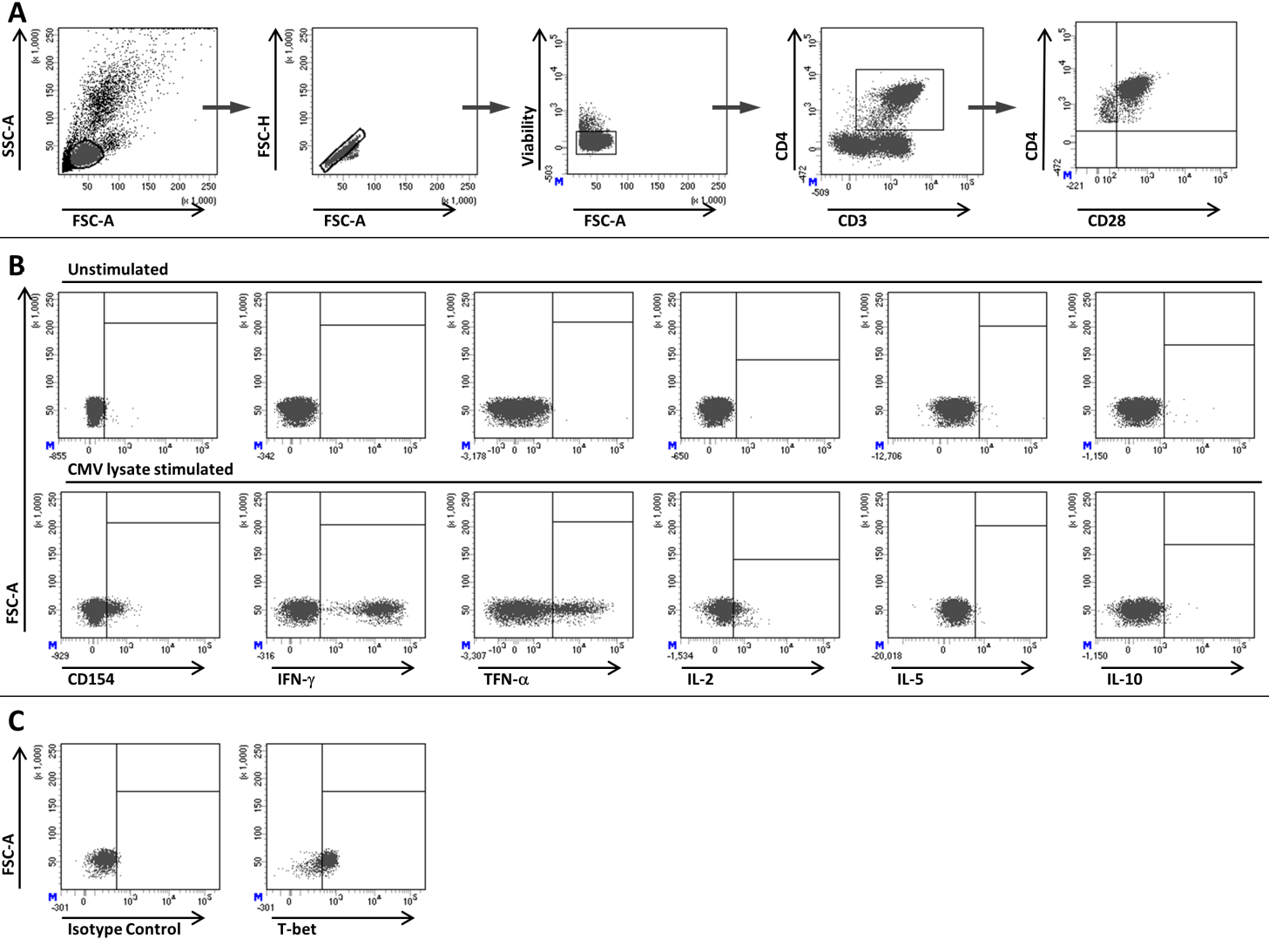


**Figure S2 Gating strategy for CMV lysate stimulated PBMC**

PBMC were stimulated with CMV lysate as detailed in the methods section. **A.** Sequential gating was performed as follows: lymphocyte gate according to forward scatter (FSC-A) and side scatter (SSC-A) parameters, single cell gate according to FSC area (FSC-A) and FSC height (FSC-H) parameters, live/dead cell gate, CD3+CD4+ gate, CD28 gate to identify CD4+CD28null and CD4+CD28+ T-cells. **B.** Cytokine and CD154 expression shown for CD4+CD28null T-cells. Gating for cytokines and CD154 expression was set based on unstimulated cells from the same patient incubated under the same conditions as CMV lysate stimulated cells. **C.** T-bet expression shown for CD4+CD28null T-cells; a fluorescence minus control that included an isotype control was employed to assist with gating.
